# Surgical productivity recovery after the COVID-19 pandemic in Japan

**DOI:** 10.3389/fpubh.2024.1302732

**Published:** 2024-02-13

**Authors:** Yoshinori Nakata, Yuichi Watanabe, Akihiko Ozaki

**Affiliations:** ^1^Teikyo University Medical Information and System Research Center, Tokyo, Japan; ^2^Teikyo University Graduate School of Public Health, Tokyo, Japan; ^3^Waseda University Graduate School of Economics, Tokyo, Japan; ^4^Department of Breast and Thyroid Surgery, Jyoban Hospital, Fukushima, Japan

**Keywords:** COVID-19, productivity change, Malmquist index, surgery, efficiency change, technical change, Japan

## Abstract

**Introduction:**

Previous studies demonstrated that the surgical productivity regressed in 2020. This study therefore explored whether the COVID-19 pandemic had any significant lasting effect of reducing the surgical productivity in Japan. This is a retrospective observational study which is an extension of the previous ones.

**Methods:**

The authors analyzed 18,805 surgical procedures performed during the study period from April 1 through September 30 in 2016–22. A non-radial and non-oriented Malmquist model under the variable returns-to-scale assumptions was employed. The decision-making unit (DMU) was defined as a surgical specialty department. Inputs were defined as (1) the number of assistants, and (2) the surgical duration. The output was defined as the surgical fee. The study period was divided into 42 one-month periods. The authors added all the inputs and outputs for each DMU during these study periods, and computed its Malmquist index, efficiency change and technical change. The outcome measures were its annual productivity, efficiency, and technical changes between the same months in each year.

**Results:**

There was no statistically significant difference in annual productivity, efficiency, and technical changes between pre-pandemic and post-pandemic periods.

**Discussion:**

No evidence was found to suggest that the COVID-19 pandemic has any significant lasting effect of reducing the surgical productivity.

## Introduction

It has been more than 3 years since the novel coronavirus disease (COVID-19) pandemic was first identified in Wuhan, China in 2019 (1, 2). In response to the COVID-19 pandemic, healthcare resources were intensively allocated to COVID-19 countermeasures, which consequently reduced the resources available for other healthcare areas, such as surgery (3). For example, a university hospital reduced the number of elective surgeries in April-May, 2020, in order to alleviate the manpower shortage in the emergency rooms for febrile patients suspected of COVID-19 infection (4). These COVID-19 countermeasures, combined with extreme restrictions on routine health services, might have led to a collapse of the entire healthcare system (5). As a result of these healthcare resources allocation shifts, the productivity progress of surgery that was unrelated to COVID-19 countermeasures suffered a negative impact from the COVID-19 pandemic in 2020 (5). Although the Japanese government declared four states of emergency in Tokyo to control the COVID-19 pandemic in 2020–2021, these states of emergency did not regress the surgical productivity (3).

Besides the states of emergency, a large number of public and hospital policies have subsequently been designed and implemented since to fight against the COVID-19 pandemic and to prevent the collapse of the healthcare system in Japan. The total effects of these policies were not yet fully investigated. In addition, it is totally unknown whether the negative impact in 2020 has any lasting influence on surgical productivity. In the present study, we investigated the lasting effects of the COVID-19 pandemic on surgical productivity changes.

We used the Malmquist model to assess surgical productivity changes in our previous studies (3, 5). Since 2000, methods of objective measurement of efficiency and productivity have made rapid advances in the fields of economics, business administration and engineering (6, 7). Data envelopment analysis (DEA) is a standard method for efficiency measurement, while the Malmquist index (MI) model is a longitudinal form of DEA. The MI model assesses productivity changes over multiple time periods, which can be decomposed into efficiency changes (ECs) and technical changes (TCs), to determine the root causes of productivity changes (8).

This study was performed to determine the surgical productivity changes in the pre- and post-pandemic periods using the MI model; it was also performed to evaluate the long-term effects of the COVID-19 pandemic on efficiency and technical changes. We hypothesized that the COVID-19 pandemic had a significant lasting effect of reducing the surgical total factor productivity.

## Materials and methods

The Institutional Review Board of Teikyo University approved a series of our studies on surgical efficiency and productivity. The need for consent was waived by the Institutional Review Board because of the retrospective observational study design. We intentionally used similar methods of data collection, analysis framework and statistical analysis because the present study is an extension of our previous ones that investigated the relationship between the COVID-19 pandemic and surgical productivity changes (3, 5).

### Data

Teikyo University Hospital is in metropolitan Tokyo, Japan, and serves ~1,000,000 individuals. It has 1,152 beds and an annual surgical volume of ~9,000 cases in 13 surgical specialties. This hospital is located in a prefecture where the state of emergency was initially declared by the Japanese government, and the hospital was acutely impacted by the COVID-19 pandemic (9). We collected data from all the surgical procedures performed in the main operating rooms of Teikyo University Hospital from April 1 through September 30 in 2020–22. We also collected those data from April 1 through September 30 in 2016–19 for use as pre-pandemic control data. Data were extracted from the Teikyo University Hospital electronic medical record system. Because of our budget and time constraints, we collected data only for 6 months (April–September) each year (3). Although there was a partial overlap of the data in 2020 and 2021 with our previous studies (3, 5), it was essential to include these data in the present study for evaluating the lasting effects of the COVID-19 pandemic on surgical productivity changes. Especially, 2022 was the year when the COVID-19 pandemic calmed down and the situation was returning to normalcy. Therefore, we partially used the same sample with our previous studies (3, 5).

The following exclusion criteria were used in this study. First, surgical procedures performed under local anesthesia by surgeons were excluded because the resource utilization for these procedures is substantially different and does not permit a clinically meaningful comparison with major surgical procedures. Oral, ophthalmic, and dermatological surgical procedures were excluded because most of these surgeries are minor procedures that can be performed under local anesthesia without anesthesiologists' involvement. In addition, procedures performed under general anesthesia in these specialties do not represent the regular activity of surgeons. Second, procedures in which the patients died within 1 month after surgery were excluded to maintain a constant quality outcome. Although patient death within 1 month after surgery does not accurately represent surgical outcome quality, it was the only available outcome measure that was common among the various surgeries analyzed in this study (8). Third, surgical procedures that were not reimbursed under the surgical payment system in 2016–22 were excluded. Finally, the surgical cases with incomplete records were excluded (3).

### Malmquist index (MI)

MI represents the dynamic productivity change of a decision-making unit (DMU) between two time periods; it is an example of comparative statics analysis (6, 7). MI is based on data envelopment analysis (DEA), which evaluates the relative efficiency of DMUs against a static efficient frontier during a single period. MI can be used to compare DEA results between two time periods, and divide the productivity change into two components: efficiency change (EC) and technical change (TC) (6). MI is defined as the product of EC and TC, where EC represents the degree of change in DMU's efficiency, while TC represents the change in efficient frontiers between the two time periods. The productivity change of a DMU between Periods 1 and 2 is mathematically represented as follows (6).


$$\begin{array}{l} \text{EC =}\\ \frac{\text{Efficiency of the DMU in Period 2 relative to the Period 2 frontier}}{\text{Efficiency of the DMU in Period 1 relative to the Period 1 frontier}}\\ \text{TC =}\\ (\frac{\text{Efficiency of the DMU in Period 1 relative to the Period 1 frontier}}{\text{Efficiency of the DMU in Period 1 relative to the Period 2 frontier}}\\ \times \;{\;\frac{\text{Efficiency of the DMU in Period 2 relative to the Period 1 frontier}}{\text{Efficiency of the DMU in Period 2 relative to the Period 2 frontier}}\;)}^{\frac{\text{1}}{\text{2}}}\\ \text{MI = EC}\times \text{TC} \end{array}$$

### Analysis framework

We used a non-radial and non-oriented Malmquist model under variable return-to-scale assumptions (6, 10). A DMU was defined as a surgical specialty department. The inputs were the number of medical doctors who assisted the surgery (assistants), as well as the duration of the surgery from skin incision to closure (surgical duration). The output was the surgical fee. Each surgical procedure was assigned a code corresponding to the surgical fee. It is classified as K000- K915 in the Japanese surgical fee schedule and is called “K codes.” Each surgical procedure is assigned to one of the K codes which correspond with surgical fees. The fee is identical regardless of who (an experienced surgeon or a surgical trainee) performs surgery if they have medical licensure, how many assistants they use, or how long it takes to complete surgery (11–14). Other fees for blood transfusion, medications, special insurance medical materials and anesthesia were excluded. The monetary values of surgical fees were originally expressed in the Japanese yen, and were converted to U.S. dollars at $1 = 140 yen to facilitate understanding by international readers.

### Comparison

The study period was divided into six 1-month periods in each year, which adds up to 42 one-month periods in our seven-year study. The sums of all inputs and outputs were used to compute MI, EC, and TC for each DMU during the 42 one-month study periods. We calculated the annual changes in productivity, efficiency, and technique between the same months in each year; these calculations were performed using DEA-Solver-Professional Software Version 15.1 (Saitech, Inc., Tokyo, Japan) (6, 10). We defined in this study the annual changes in 2016–17, 2017–18, and 2018–19 as “pre-pandemic,” while the annual changes in 2019–20, 2020–21, and 2021–22 as “post-pandemic.” We focused on the total effects of the pandemic on the surgical productivity because the short-term effects had already been reported in our previous papers (3, 5). Therefore, we aggregated the post-pandemic data and compared them to the pre-pandemic ones.

All surgical departments in the sample were assigned MI, EC, and TC values; their natural logarithms were calculated to allow interpretation as percent changes (10, 15, 16). Natural logarithm of MI values greater than, equal to, and less than 0 indicated increased, unchanged, and decreased productivity from Period 1 to 2, respectively. Similarly, natural logarithms of EC and TC > 0 indicated efficiency and technical improvements, respectively. The natural logarithm of MI equals the sum of natural logarithms of TC and EC (10, 16).

The following 10 surgical specialty departments were included in our analysis; cardiovascular surgery, emergency surgery, general surgery, neurosurgery, obstetrics & gynecology, orthopedics, otolaryngology, plastic surgery, thoracic surgery, and urology. The natural logarithms of MI, EC, and TC were obtained for each surgical specialty department according to corresponding 1-month periods during the study period; their means and standard errors were calculated (16).

### Statistical analysis

Excel Statistics Software (Social Survey Research Information Co., Ltd., Tokyo, Japan) was used for statistical analysis. The natural logarithms of MIs, ECs, and TCs for surgical specialties were compared between pre-pandemic and post-pandemic periods, and those of each period against 0 using *t*-tests. *P*-values < 0.05 were considered statistically significant (16, 17).

## Results

We analyzed 18,805 surgical procedures performed from April 1 through September 30 in 2016–22. The characteristics of surgery are shown in Tables 1, 2. The total surgical volume per year has decreased by 11% in the post-pandemic period compared to the pre-pandemic period.

**Table 1:** Characteristics of surgery 2016–2019 (pre-pandemic period).

**Specialty**	**Cases/year**	**Assistants/case**	**Surgical duration/case (min)**	**Fee/case (US dollars)**
Cardiovascular surgery	245 (28)	2.86	214	6,177
Emergency surgery	342 (77)	1.75	129	2,148
General surgery	526 (24)	2.16	191	2,896
Neurosurgery	159 (22)	1.77	190	5,868
Obstetrics and gynecology	351 (36)	2.01	103	2,022
Orthopedics	508 (26)	2.41	118	2,164
Otolaryngology	185 (4)	1.49	116	1,592
Plastic surgery	137 (1)	1.54	108	1,412
Thoracic surgery	114 (2)	1.94	142	4,283
Urology	253 (5)	1.73	119	2,148
All surgical procedures	2,821 (177)	2.06	144	2,848

The numbers in the parenthesis in cases/year are the average number of surgical procedures per year that were performed outside the regular hospital hours, including late nights and holidays.

Assistants/case, surgical duration/case, and fee/case are expressed in means.

The monetary values of surgical fees were converted to U.S. dollars at $1 = 140 yen.

**Table 2:** Characteristics of surgery 2020–2022 (post-pandemic period).

**Specialty**	**Cases/year**	**Assistants/case**	**Surgical duration/case (min)**	**Fee/case (US dollars)**
Cardiovascular surgery	256 (20)	3.43	162	5,214
Emergency surgery	344 (50)	2.37	118	2,196
General surgery	485 (13)	2.48	192	3,008
Neurosurgery	102 (11)	2.12	192	6,264
Obstetrics and gynecology	312 (28)	2.08	119	2,105
Orthopedics	430 (25)	2.73	138	2,593
Otolaryngology	130 (2)	1.42	123	1,737
Plastic surgery	104 (0)	2.29	145	1,896
Thoracic surgery	96 (0)	2.83	176	4,243
Urology	249 (6)	2.12	121	2,920
All surgical procedures	2,508 (159)	2.45	147	2,920

The numbers in the parenthesis in cases/year are the average number of surgical procedures per year that were performed outside the regular hospital hours, including late nights and holidays.

Assistants/case, surgical duration/case and fee/case are expressed in means.

The monetary values of surgical fees were converted to U.S. dollars at $1 = 140 yen.

The natural logarithms of MIs (percent productivity changes) were shown in Table 3. The productivity change of all surgical procedures was not significantly different from zero, which means that there was no productivity change either during pre-pandemic or during post-pandemic period. There was no statistically significant difference in overall productivity change between pre-pandemic and post-pandemic periods. The subgroup analysis demonstrated that obstetrics & gynecology and thoracic surgery had significantly different productivity changes between pre-pandemic and post-pandemic periods (*p* = 0.0015 and *p* = 0.0057, respectively).

**Table 3:** Percent productivity changes.

**Specialty**	**Pre-pandemic**	**Post-pandemic**
Cardiovascular surgery	+1.8 ± 9.2	−0.5 ± 5.1
Emergency surgery	+5.6 ± 4.8	−3.1 ± 6.6
General surgery	+2.2 ± 10.5	+2.5 ± 9.1
Neurosurgery	−1.8 ± 5.5	−0.2 ± 9.1
Obstetrics and gynecology^**^	+4.4 ± 2.2	−6.3 ± 2.1^*^
Orthopedics	−2.1 ± 8.2	+1.6 ± 7.8
Otolaryngology	+6.0 ± 4.7	+13.4 ± 18.9
Plastic surgery	−6.0 ± 20.1	−19.5 ± 16.8
Thoracic surgery^**^	−27.5 ± 8.0^*^	+8.2 ± 9.1
Urology	+6.4 ± 7.0	−9.5 ± 5.4
All surgical procedures	−1.1 ± 2.9	−1.3 ± 3.2

The values are expressed as mean ± SE.

^*^Indicates that the value is significantly different from 0 (p < 0.05).

^**^Indicates that there was a statistically significant difference between “pre-pandemic” and “post-pandemic” (p < 0.05).

The natural logarithms of ECs (percent efficiency changes) were shown in Table 4. The efficiency change of all surgical procedures was not significantly different from zero, which means that there was no efficiency change either during pre-pandemic or during post-pandemic period. There was no statistically significant difference in overall efficiency change between pre-pandemic and post-pandemic periods. The subgroup analysis demonstrated that there were no surgical specialties that had different efficiency changes between pre-pandemic and post-pandemic periods.

**Table 4:** Percent efficiency changes.

**Specialty**	**Pre-pandemic**	**Post-pandemic**
Cardiovascular surgery	+1.4 ± 8.4	+5.0 ± 4.3
Emergency surgery	+8.8 ± 5.7	+1.5 ± 9.0
General surgery	−6.8 ± 13.1	+4.9 ± 12.6
Neurosurgery	+3.6 ± 5.6	+5.0 ± 8.1
Obstetrics and gynecology	+8.2 ± 4.2	−5.2 ± 7.2
Orthopedics	−0.2 ± 12.3	+7.1 ± 8.9
Otolaryngology	+23.2 ± 8.8^*^	+10.7 ± 17.3
Plastic surgery	+26.1 ± 18.5	−25.7 ± 21.3
Thoracic surgery	−19.3 ± 8.0^*^	+3.9 ± 12.0
Urology	+17.3 ± 10.2	−9.5 ± 9.3
All surgical procedures	+6.2 ± 3.3	−0.2 ± 3.8

The values are expressed as mean ± SE.

^*^Indicates that the value is significantly different from 0 (p < 0.05). There was no statistically significant difference between “pre-pandemic” and “post-pandemic.”

The natural logarithms of TCs (percent technical changes) were shown in Table 5. The technique of all surgical procedures regressed significantly during the pre-pandemic period (*p* = 0.0005). It regressed by 7.4% on average during the pre-pandemic period. However, there was no statistically significant difference in overall technical change between pre-pandemic and post-pandemic periods. The subgroup analysis demonstrated that there were no surgical specialties that had different technical changes between pre-pandemic and post-pandemic periods.

**Table 5:** Percent technical changes.

**Specialty**	**Pre-pandemic**	**Post-pandemic**
Cardiovascular surgery	+0.1 ± 3.7	−5.4 ± 3.0
Emergency surgery	−3.9 ± 3.7	−4.6 ± 6.3
General surgery	+7.0 ± 10.6	−2.4 ± 9.4
Neurosurgery	−4.6 ± 3.7	−5.3 ± 2.7
Obstetrics and gynecology	−3.9 ± 4.1	−1.1 ± 7.5
Orthopedics	−8.0 ± 9.7	−5.5 ± 5.1
Otolaryngology	−16.3 ± 8.1	+2.7 ± 20.4
Plastic surgery	−25.4 ± 9.2^*^	+6.3 ± 18.1
Thoracic surgery	−8.3 ± 4.0	+4.3 ± 10.6
Urology	−10.6 ± 6.1	0.0 ± 7.9
All surgical procedures	−7.4 ± 2.1^*^	−1.1 ± 3.0

The values are expressed as mean ± SE.

^*^Indicates that the value is significantly different from 0 (p < 0.05). There was no statistically significant difference between “pre-pandemic” and “post-pandemic.”

## Discussion

On the contrary to our hypothesis presented in Introduction, no evidence was found to suggest that the COVID-19 pandemic has any significant lasting effect of reducing the surgical productivity. There was no statistically significant difference in productivity change between pre-pandemic and post-pandemic periods. In other words, the surgical productivity has fully recovered to the pre-pandemic level by 2022 despite a short-term productivity regress in 2020 (5). In addition, our subgroup analysis demonstrated that there were no surgical specialties, except obstetrics & gynecology and thoracic surgery, that had different productivity, efficiency or technical changes between pre-pandemic and post-pandemic periods.

Although the findings above may appear similar to our previous results (3), their scientific contribution is totally different. In the previous study (3), we compared ten-day time periods which included or excluded the states of emergency in short study period. In contrast, we compared the same month in the consecutive years in pre- and post-pandemic periods over seven years in the present study. Our previous study focused only on the effects of states of emergency (3), while the comparison in the present study included all the relevant public and hospital policies that were designed and implemented in addition to the states of emergency. This study has revealed the total effects of all public and hospital policies on annual surgical productivity, efficiency and technical changes between pre-pandemic and post-pandemic periods, which were unknown from the previous study (3). This is the first study in Japan to elucidate the lasting effects of the COVID-19 pandemic on annual surgical productivity changes by using 7-year actual surgical data.

The reason for the findings above is impossible to identify from the present study because many public and hospital policies have changed during the pandemic. The first state of emergency started in April and ended in May 2020 in Tokyo. In response to this state of emergency, Teikyo University Hospital limited the number of elective surgeries on April 6, but removed all the limitation on May 25, 2020. It has not restricted the number of elective surgeries since. Although this change may have reduced the surgical productivity in the short term (5), it had no effects on surgical productivity for our study period of 2016–22.

There were some surgical specialties that showed significant changes in productivity, efficiency, and technique during the study periods. For example, obstetrics & gynecology significantly reduced productivity during the post-pandemic period (*p* = 0.0092), while thoracic surgery reduced productivity during the pre-pandemic period (*p* = 0.0031). It is also difficult to specify any causes for these changes from the present study. However, our hospital appointed new chief surgeons in these two surgical departments during our study period, which might have affected their productivity changes. In addition, the number of obstetrics & gynecology and thoracic surgery cases decreased after the pandemic by 10–15%. No other surgical specialties had significantly different productivity, efficiency or technical changes between pre-pandemic and post-pandemic periods.

It is well-known that large Japanese hospitals with more than 400 beds actively perform after-hours surgeries and other procedures during the pandemic (18). However, judging from the data shown Tables 1, 2 on the average number of surgical procedures that were performed outside the regular hospital hours, no differences in the ratio of after-hour surgeries before and after the pandemic were noted. Teikyo University Hospital had already been performing a significant number of after-hour surgeries (6.3%) before the pandemic, and did not more actively perform them (6.4%) in the post-pandemic period.

Since the present study is an extension of our previous ones (3, 5), it has similar methodological limitations. First, we could not exclude the effects of the revisions in the nation-wide fee schedules from our analysis in productivity changes. There were three revisions on April 1 in 2018, 2020, and 2022 during our study period (12–14). However, our previous study demonstrated that the effects of revisions were insignificant in productivity change (16). We can assume that these revisions had a minimal impact on our conclusions without seriously biasing our analysis. Second, our results in the present study may be false negative. We analyzed 18,805 surgical procedures over 7 years, which is expected to be a sufficiently large sample. The power analysis demonstrated that the possibility of type II errors in our sample was 0.0072 when we fail to reject the null hypothesis that the post-pandemic percent productivity change of all the surgical procedures equals zero (19). Therefore, the possibility of type II errors is small enough although we cannot completely exclude the possibility of false negative results. Third, generalization may be impossible because this study was conducted in a single large teaching hospital in Tokyo. Specifically, the generalizability of our findings is limited because of our focus on a single country and a specific healthcare context. However, Tokyo was one of the most impacted areas by the COVID-19 pandemic in Japan (9). If our hospital in Tokyo fully recovered its surgical productivity from the COVID-19 pandemic, other hospitals in Japan should also have recovered. In addition, there is an advantage to studying surgical productivity in a single hospital. Since one of the significant resource inputs is ancillary services such as operating room nursing practices, all these factors are held constant in a single hospital. By analyzing surgical departments in the same hospital, they all face the same systemic advantages and disadvantages of those services (20). Fourth, the study's focus on Japan is narrow in scope. It would greatly enhance the relevance of our findings if comparisons were made with other hospitals within Japan or with hospitals in other countries. However, the data were unavailable for us.

## Conclusion

In conclusion, no evidence was found to suggest that the COVID-19 pandemic has any significant lasting effect of reducing the surgical productivity in Japan. The overall productivity, efficiency, and technique did not significantly differ between pre- and post-pandemic periods. Kumagai showed that after the second state of emergency declaration, the decline in the number of physician visits caused by the spread reduced by almost half, and that the staying-at-home effect did not persist (21). These findings suggest that the surgical productivity has recovered to the pre-pandemic level by 2022 despite a short-term productivity regress in 2020. Our findings would be one of the valuable lessons learned from our experience of the COVID-19 pandemic. Based on our findings for healthcare policy and surgical practice, we can better deal with the pandemic if the similar one hits us again in the future. A comparative analysis with other hospitals or countries could provide a more comprehensive understanding of the pandemic's impact on surgical productivity.

## Transparency statement

There was a partial overlap of the data with our previous studies. It was essential to include these data in the present study for evaluating the long-term effects of the COVID-19 pandemic on surgical productivity changes.

## Author's note

This study was presented in part on June 5, 2023 at Euroanesthesia 2023 Meeting of European Society of Anaesthesiology and Intensive Care held in Glasgow, UK.

## Data availability statement

The original contributions presented in the study are included in the article/supplementary material, further inquiries can be directed to the corresponding author.

## Ethics statement

The studies involving humans were approved by Teikyo University Institutional Review Board. The studies were conducted in accordance with the local legislation and institutional requirements. Written informed consent for participation was not required from the participants or the participants' legal guardians/next of kin in accordance with the national legislation and institutional requirements.

## Author contributions

YN: Conceptualization, Funding acquisition, Investigation, Methodology, Project administration, Supervision, Validation, Writing – original draft, Writing – review & editing. YW: Data curation, Formal analysis, Software, Writing – review & editing. AO: Supervision, Validation, Writing – review & editing.
